# Clinical application value of metagenomic next-generation sequencing in the diagnosis of spinal infections and its impact on clinical outcomes

**DOI:** 10.3389/fcimb.2023.1076525

**Published:** 2023-02-08

**Authors:** Guang Zhang, Hongqi Zhang, XiaoJiang Hu, Dongcheng Xu, Bo Tang, Mingxing Tang, Shaohua Liu, Yanbing Li, Wen Xu, Chaofeng Guo, Qile Gao

**Affiliations:** ^1^ Department of Spine Surgery and Orthopaedics, Xiangya Hospital, Central South University, Changsha, China; ^2^ Department of Clinical Laboratory, Xiangya Hospital, Central South University, Changsha, China; ^3^ National Clinical Research Center for Geriatric Disorders, Xiangya Hospital, Central South University, Changsha, China; ^4^ Department of Scientific Affaires,Guangzhou Sagene Biotechnology Company, Limited, Guangzhou, China

**Keywords:** metagenomic next-generation sequencing, diagnosis, spinal infection, traditional, prognosis

## Abstract

This study aimed to evaluate the impact of precise treatment administered according to the results of metagenomic next-generation sequencing (mNGS) on the clinical outcomes of patients with spinal infections. In this multicenter retrospective study, the clinical data of 158 patients with spinal infections who were admitted to Xiangya Hospital Central South University, Xiangya Boai Rehabilitation Hospital, The First Hospital of Changsha, and Hunan Chest Hospital from 2017 to 2022 were reviewed. Among these 158 patients, 80 patients were treated with targeted antibiotics according to the mNGS results and were assigned to the targeted medicine (TM) group. The remaining 78 patients with negative mNGS results and those without mNGS and negative microbial culture results were treated with empirical antibiotics and assigned to the empirical drug (EM) group. The impact of targeted antibiotics based on the mNGS results on the clinical outcomes of patients with spinal infections in the two groups was analyzed. The positive rate of mNGS for diagnosing spinal infections was significantly higher than that of microbiological culture (*X* ^2^=83.92, *P*<0.001), procalcitonin (*X* ^2^=44.34, *P*<0.001), white blood cells (*X* ^2^=89.21, *P* < 0.001), and IGRAs (Interferon-gamma Release Tests) (*X* ^2^ = 41.50, *P* < 0.001). After surgery, C-reactive protein (CRP) and erythrocyte sedimentation rate (ESR) showed a decreasing trend in the patients with spinal infections in both the TM and EM groups. The decrease in CRP was more obvious in the TM group than in the EM group at 7, 14 days, 3, and 6 months after surgery (*P*<0.05). The decrease in ESR was also significantly obvious in the TM group compared with the EM group at 1 and 6 months after surgery (*P*<0.05). The time taken for CRP and ESR to return to normal in the TM group was significantly shorter than that in the EM group (*P*<0.05). There was no significant difference in the incidence of poor postoperative outcomes between the two groups. The positive rate of mNGS for the diagnosis of spinal infection is significantly higher than that of traditional detection methods. The use of targeted antibiotics based on mNGS results could enable patients with spinal infections to achieve a faster clinical cure.

## 1 Introduction

Spinal infections include spondylitis (vertebral osteomyelitis), suppurative spondylitis, sepsis, and epidural abscesses. With the rapid growth of the aging population and the widespread adoption of advanced medical imaging techniques, the incidence and diagnosis rates of spinal infections have been increasing in recent years, but spinal infections are relatively rare and challenging infectious diseases (Duarte and Vaccaro, 2013). Early, rapid and accurate diagnosis and targeted anti-infective therapy for spinal infections remain the huge challenges faced by physicians.

Rapid and accurate identification of infectious pathogens and the adoption of pathogen-targeted antibiotics have always been the focus and difficulties in the diagnosis and treatment of spinal infections (Kim et al., 2012). At present, due to the lack of specificity of the symptoms and signs in patients with spinal infections, the preoperative diagnosis of spinal infections is mainly based on comprehensive judgments made by spine surgeons according to the results of peripheral blood tests and imaging examinations, which may lead to apparent delay in diagnosis and undoubtedly result in the spread of infections, thus causing the occurrence of severe complications (Berbari et al, 2015). In terms of imaging examinations, although imaging techniques are constantly developing and the diagnosis rate of spinal infections is increasing, imaging examinations cannot provide the basis for the etiological diagnosis of spinal infections (Tali et al., 2015). The erythrocyte sedimentation rate (ESR), C-reactive protein (CRP) and procalcitonin (PCT) are sensitive markers of infection, but they also lack specificity for the diagnosis of spinal infections (Lee et al., 2019). A previous study showed that microbiological culture had a low sensitivity and failed to meet the clinical needs for the diagnosis of spinal infections (Ma et al., 2022). Additionally, X-pert is mainly used to diagnose spinal tuberculosis (TB) in clinical practice and has good sensitivity and specificity, but X-pert is limited to the diagnosis of spinal TB because it cannot identify microorganisms other than *Mycobacterium tuberculosis* (Furin et al., 2019). Therefore, there is an urgent need for a novel detection technology with high specificity, high sensitivity and low time consumption for the clinical diagnosis of spinal infections, and these methods should not only identify the pathogens of spinal infections but also can be applied to guide precision treatment.

Metagenomic next-generation sequencing (mNGS) is a cutting-edge technology that has emerged in recent years. It has been reported that mNGS can theoretically detect all known and unknown pathogens in samples and has been used in the diagnosis of atypical and rare infections (Han et al., 2019). Previous case reports and clinical studies have shown that mNGS has been applied to various sample types. A major advantage of mNGS over traditional diagnostic methods is that mNGS can diagnose complex infectious diseases (Gu et al., 2021). In recent years, a number of studies have reported the clinical application of mNGS in the diagnosis and treatment of multiple infectious diseases (Miao et al., 2018; Zhang et al., 2019; Fu et al., 2021). At present, mNGS has been gradually applied in the detection of pathogens of spinal infections in clinical practice, which can help spine surgeons determine early and precise treatment options (Ma et al., 2022). However, few reports describe the use of mNGS for pathogen detection in patients with spinal infections, and the influence of mNGS results on patient outcomes is unknown. Therefore, we tried to use mNGS to detect the pathogens of spinal infection, and we found that the detection rate was high. According to the mNGS detection results, targeted medication, and the achievement of satisfactory results, we conducted a multicenter retrospective study of 158 patients with spinal infections, and evaluated the influence of targeted antibiotics selected according to the mNGS results on the prognosis of patients to provide a clinical basis for the application of mNGS in spinal infections.

## 2 Methods

### 2.1 Study patients

A total of 158 patients with spinal infections and who were admitted to Xiangya Hospital of Central South University, Xiangya Fraternity Rehabilitation Hospital, Changsha First Hospital and Hunan Chest Hospital from 2017 to 2022 were enrolled.

The inclusion criteria were as follows: patients with nodular granulomatous inflammation, suppurative inflammation, or infectious lesions on pathological examination.

The exclusion criteria were as follows: (1) patients with spinal tumors by pathological examination; (2) patients with spinal bone destruction whose lesions were normal tissue or aseptic inflammation by pathological examination; (3) spinal infection patients whose mNGS test results were inconsistent with the microbial culture results; and (4) patients who were not willing to undergo mNGS detection but who had a positive microbial culture.

This study was approved by the Ethics Committee of Xiangya Hospital Central South University, and written informed consent was obtained from all patients.

### 2.2 Study design

The four hospital statistics since 2017 that were evaluated included spinal bone destruction in the hospital and surgical treatment of the patients with spinal open surgery, the samples used in our study were the most typical suppurative soft tissue of the lesions selected for open spinal surgery, and suppurative soft tissue from spinal lesions was obtained during surgery and immediately stored in sterile culture tubes for examination(normal line cultivation, anaerobic and aerobic cultivation), and the same procedure was applied to all samples. For patients who were highly suspected to have spinal tuberculosis by preoperative imaging examination and serological examination, intraoperative tissue samples were also taken for X-pert examination and mycobacterial culture, and the patients voluntarily decided whether to have intraoperative samples taken for mNGS detection). Excluding patients with spinal tumors and aseptic inflammation on pathological examination, the postoperative pathological examination results of all the patients included in the study showed suppurative inflammation, nodular granulomatous inflammation, or infectious lesions. Culture-positive cases without mNGS and those whose mNGS test results were inconsistent with culture results were not included in this study. Patients who tested positive for mNGS and consistent with microbial culture results and those who only tested positive for mNGS were included in the targeted drug delivery group, for a total of 80 patients (The mNGS results were consistent with the culture results in 18 patients, and the culture results were negative in the remaining 62 patients), and antibiotics were used according to the examination results after surgery. The patients with negative mNGS test results and microbial culture results and the patients who were unwilling to undergo mNGS tests and negative microbial culture results were included in the empirical medication group, for a total of 78 patients (Among them, there were 12 cases with negative mNGS test results and negative microbial culture results, and 66 cases without mNGS and negative microbial culture results). After the operation, empirical medication was administered according to the local epidemiological characteristics, serological examination and imaging examination. The specific treatment is displayed in the **Supplement Data Table**. The effect of empirical medication was evaluated according to the improvement of postoperative symptoms and regular serological tests (CRP, ESR, etc.), and the use of antibiotics was adjusted in time. The two groups of patients had regular follow-up of blood sedimentation and C-reactive protein levels after surgery at 7 days, 14 days after the operation, 1 month, 3 months after the operation, and 6 months and 1 year after surgery, and there was follow-up of the patient prognosis (Poor prognosis was defined as poor wound healing, loose internal fixation or fracture, reinfection of the same surgical site, more than two debridement operations, death, etc.). The criteria for a clinical cure were the return of inflammatory indicators, such as the erythrocyte sedimentation rate (ESR) and CRP, to normal, relief of local symptoms, and no postoperative infection-related complications.

**Figure 1:**
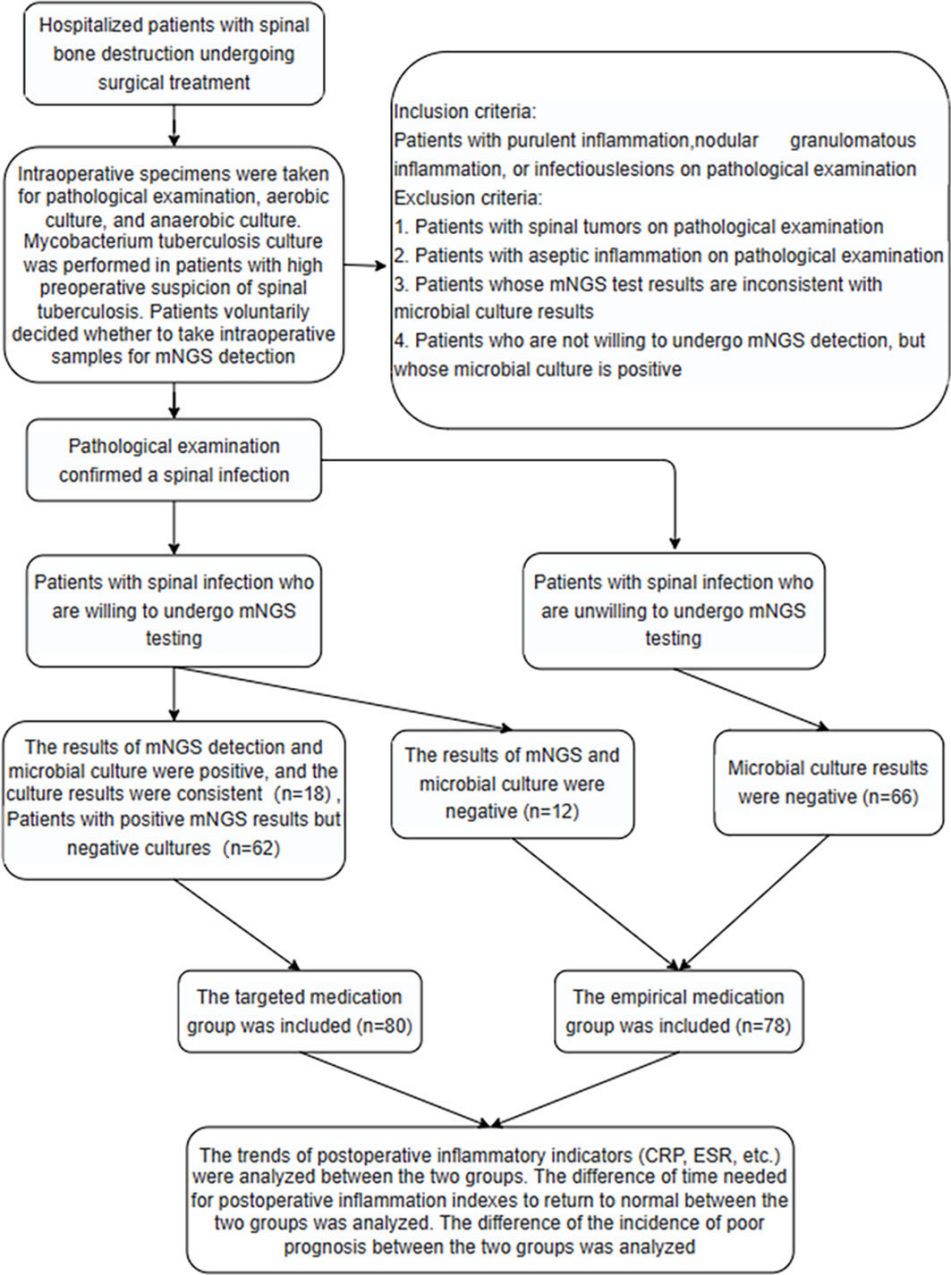
Study flowchart.

The clinical data of the enrolled patients were collected in detail, and the positive rate of mNGS and PCT, WBC, IGRAs and other traditional methods in the diagnosis of spinal infection were compared. The positive concordant percentage and negative concordant percentage of mNGS and tissue microbial culture in the diagnosis of spinal infection pathogens were compared. The detection rate of MTB in the spinal column was compared between mNGS, X-pert and microbial culture. The change trend of ΔCRP (pre-op CRP-post-op CRP) and ΔESR (pre-op ESR-post-op ESR) were compared between the two groups at different time points. The time required for C-reactive protein and erythrocyte sedimentation rate to return to the normal range was compared between the two groups, and the incidence of poor prognosis was compared between the two groups (**Figure 1**).

### 2.3 mNGS procedures and analyses

#### 2.3.1 Experimental methods

(1) Sample processing and nucleic acid extraction

Samples were stored in liquid nitrogen before testing. Chop the tissue and transfer to a centrifuge tube. 0.5mL trypsin was added, and the mixture was shaken and mixed for 5min. A metal bath at 37°C was used for 30min. 400ul of the treated sample was mixed with glass beads, and the wall was broken by biological specimen homogenizer (bioprep-24, tien). Magnetic bead method (MAGEN Guangzhou, CHINA) was used to extract and purify microbial DNA from the broken wall specimens. Prior to library preparation, the concentration of extracted nucleic acids was measured using a Qubit4 (Thermo Fisher) fluorometer.

(2) Library construction

The extracted nucleic acid was used to construct the metagenomic Library according to the instructions of the Library construction Kit Nextera XT DNA Library Prep Kit (Illumina, USA). Firstly, DNA fragmentation was carried out, then end repair and joint joining were carried out, and finally Library amplification was carried out. After the construction of the library meeting the requirements of sequencing (library size: ~300bp). Libraries were assessed by quality assessment using a Qubit 4 fluorometer (Thermofisher) and agarose gel electrophoresis. Eligible libraries with different index adaptor sequences were mixed in equal volumes and high-throughput sequencing was performed using the Illumina Nextseq 550 DX sequencing platform (sequencing strategy: SE75) (Nextseq 550 DX is an FDA-approved and CE-IVD-certified sequencer).

#### 2.3.2 Production and communication analysis process

The dismounted data were basically filtered by FastQC software to obtain high-quality clean reads, that is, sequences with connectors, low-quality bases and too short (< 50bp) were removed. Clean reads were paired with human genome reference sequences using BowtiE2 (version: GRCh38) was used for comparison, and host-related reads were removed. The remaining reads were compared with the specific pathogenic microorganism database (containing 18000+ medical microorganism information) optimized by Guangzhou Sage for BWA analysis, and the number of detected sequences (reads) of pathogenic microorganisms was obtained.

#### 2.3.3 Interpretation criteria of positive results

After obtaining credible analysis data, the report is interpreted, and the interpretation criteria are as follows:

(1) Report if 1 Mycobacterium tuberculosis is detected;
(2) As for the virus, more than 3 viruses are detected in different regions, that is, the report;
(3) For bacteria other than mycobacteria, viruses and parasites, the top 10 bacteria in each category were selected, and the suspected pathogens were reported based on clinical characteristics.

### 2.4 Statistical analysis

All statistical analyses were carried out using SPSS 25.0 software. Continuous variables such as age and time are expressed as the mean ± standard deviation (SD). Comparisons between the groups were performed using one-way analysis of variance (ANOVA). The differences in sex ratio between the two groups were analyzed using the chi-squared test. The differences in the positive rates between mNGS and traditional microbiological culture, PCT, WBC, and IGRAs were analyzed using the chi-square test. A nonparametric test was used to analyze the differences in the changes in CRP and ESR between the two groups before surgery and at different time points after surgery, as well as the differences between the groups in the time taken for CRP and ESR to return to normal after surgery. Fisher's exact test was used to analyze the differences in the incidence of poor outcomes in the patients between the two groups. The graphs were generated using GraphPad Prism 9 and Microsoft Excel.

## 3 Results

### 3.1 Patient characteristics

A total of 158 patients with spinal infections were enrolled in this study, including 81 males and 77 females. A total of 80 patients received targeted anti-infective therapy according to their mNGS results after the operation, and these patients included 46 males and 34 females, with an average age of 54.91±15.81 years. The remaining 78 patients with an unclear etiology were treated with empirical medication after operation, and these patients included 35 males and 43 females, with an average age of 52.28±16.02 years. There was no significant difference in age or sex between the two groups (**Table 1**).

**Table 1:** The comparison of the difference of ΔCRP (pre-op CRP-post-op CRP) and ΔESR (pre-op ESR-post-op ESR) [*M*(*P*_25_, *P*_75_)] between two groups at different follow-up times.

Project	Targeted medication group (n=80)	Empirical medication group (n=78)	*Z*-absolute value	*P*-value
Age(years) *	54.91±15.81	52.28±16.02		0.301
Female/Male †	34/46	43/35		0.112
Δ CRP(mg/L)‡				
7days post-op	6.63(-9.17,36.83)	-9.87(-26.10,0.75)	4.061	0.000
14days post-op	15.91(0.60,54.30)	-0.42(-6.68,18.80)	2.233	0.026
1 mo post-op	32.97(7.39,69.20)	0.61(-4.17,49.05)	1.336	0.181
3 mo post-op	23.10(8.56,64.22)	3.99(-0.38,17.30)	2.726	0.006
6 mo post-op	17.50(3.44,78.83)	2.30(-4.61,17.36)	2.534	0.011
1 ye post-op	12.41(1.67,81.07)	9.82(0.31,18.57)	0.568	0.570
Δ ESR(mm/h)‡				
7 days post-op	11.00(-17.00,43.00)	10.00(-13.75,40.50)	0.069	0.945
14 days post-op	19.00(-9.75,42.75)	0.00(-21.50,22.50)	1.920	0.055
1 mo post-op	48.50(22.50,83.75)	-10.00(-20.00,28.00)	2.301	0.021
3 mo post-op	44.00(23.25,87.75)	31.00(12.75,58.25)	1.623	0.105
6 mo post-op	47.50(34.75,83.50)	27.00(-11.50,61.50)	2.105	0.035
1 ye post-op	40.00(15.50,64.50)	55.00(34.75,77.50)	0.816	0.415

Δ CRP=Pre-op CRP – Post-op CRP; Δ ESR= Pre-op ESR – Post-op ESR.

* one-way ANOVA: F = 1.079, p = 0.301; ANOVA: Analysis of variance.

† one-way Pearson’s chi-square test: x^2^=2.521, p = 0.112.

‡ one-way Wilcoxon rank-sum test.

### 3.2 Analysis of mNGS and microbial culture detection results

**Figure 2:**
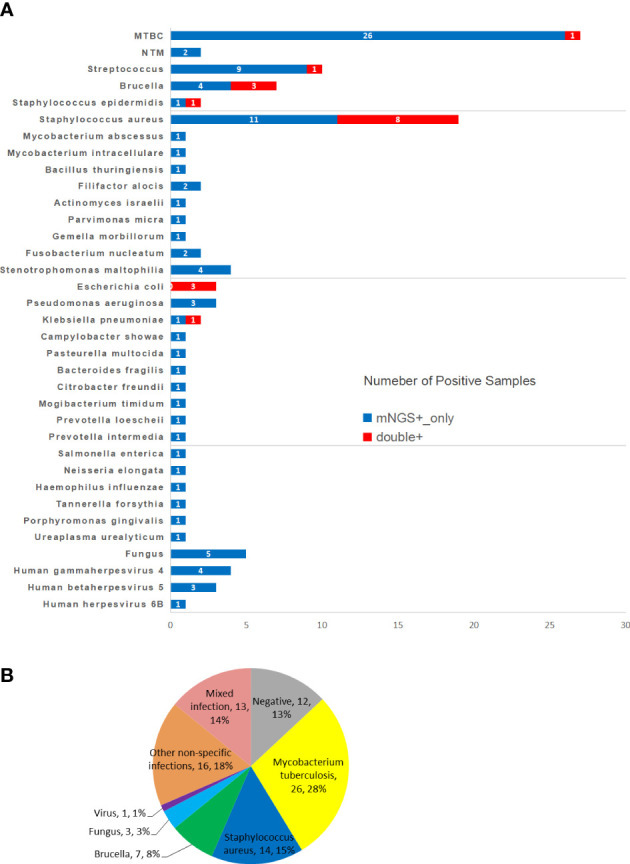
Positive overlap between metagenomic next-generation sequencing (mNGS) and culture cultures in spinal infected lesions, pathogen species and frequencies detected by both methods are plotted in histogram **(A)**. **(B)** Pie chart shows the classification of mNGS detection results for 92 spinal infection patient samples. mNGS, metagenomic next-generation sequencing; MTBC, *Mycobacterium Tuberculosis Complex*; NTM, nontuberculous mycobacteria.

In this study, a total of 92 surgical specimens were detected by mNGS, among which 80 specimens were positive (67 samples with only one pathogen and 13 samples with multiple pathogens). The common pathogens of spinal infections included *Mycobacterium tuberculosis* (27 cases, a single pathogen infection in 26 cases, mixed infection in 1 case), *Staphylococcus aureus* (19 cases, a single pathogen infection in 14 cases, mixed infection in 5 cases), Brucella (7 cases, all were infected with a single pathogen), Streptococcus (10 cases, a single pathogen infection in 6 cases, mixed infection in 4 cases), *Escherichia coli* (3 cases, a single pathogen infection in 1 case, mixed infection in 2 cases), fungi (5 cases, a single pathogen infection in 3 cases, mixed infection in 2 cases), and viruses (8 cases, a single pathogen infection in 1 case, mixed infection in 7 cases). The positive rate of microbial culture in detecting Staphylococcus, Brucella and Escherichia coli was high, but the positive detection rate of other pathogens of spinal infection was low. However, the detection rate of mNGS was higher than that of microbial culture in detecting both common and rare pathogens of spinal infection (**Figure 2A**). Analysis of the mNGS detection results of the independent samples from each patient showed that the pathogen detection rates were as follows: *Mycobacterium tuberculosis* (26/92, 28%), *Staphylococcus aureus* (14/92, 15%), spinal coinfection (13/92, 14%), Brucella (7/92, 8%), other nonspecific infectious pathogens (16/92, 18%), fungi (3/92, 3%), and viruses (1/92, 1%) (**Figure 2B**).

### 3.3 Comparison of the positive rate of mNGS and several traditional detection methods

**Figure 3:**
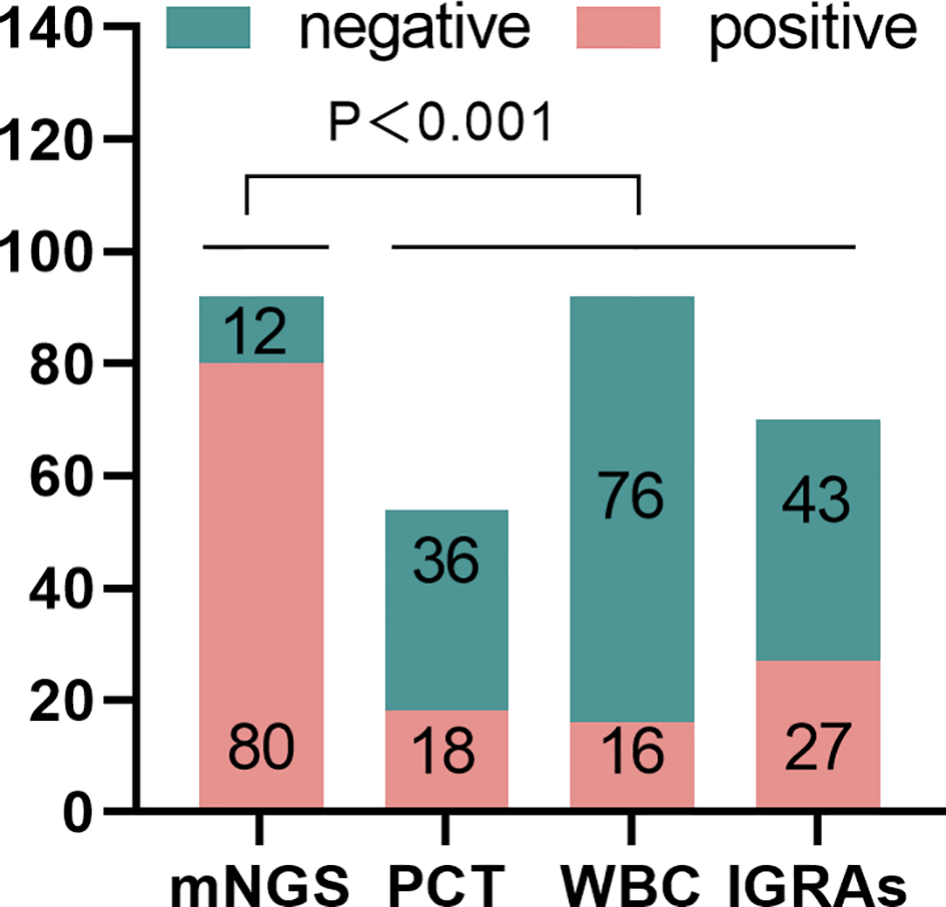
The positive rate of metagenomic next-generation sequencing (mNGS)for spinal infection pathogens was significantly higher than that of PCT, WBC, and IGRAs (*P* < 0.001).PCT, procalcitonin; IGRAs, Interferon-gamma Release Tests.

The positive rates of mNGS, PCT, WBC and IGRAs were 82/92, 86.32%, 18/54, 30.33%, 16/92, 17.39% and 27/70, 38.57%, respectively. The positive rate of mNGS was higher than that of PCT (*X* ^2^=44.34, *P* < 0.001), WBC (*X* ^2^=89.21, *P* < 0.001), and IGRAs (*X* ^2^=41.50, *P* < 0.001) (**Table 2**; **Figure 3**).

**Table 2:** Comparison of positive rate between mNGS and traditional detection methods.

Test methods	Positive	Negative	Total	Positive rate (%)	*X* ^2^ value	*P*-value
mNGS	80	12	92	86.96		
culture	18	74	92	19.57	83.92	0.000*
PCT	18	36	54	30.33	44.34	0.000*
WBC	16	76	92	17.39	89.21	0.000*
IGRAs	27	43	70	38.57	41.50	0.000*

* indicates that Pearson's chi-square test was used to compare the sensitivity between mNGS and other traditional detection methods. Positive PCT and WBC indicate increased PCT levels and WBC count; negative PCT and WBC indicate normal PCT levels and WBC count.

### 3.4 Comparison of the positive rate of mNGS detection and microbial culture and consistency analysis of detection results

**Figure 4:**
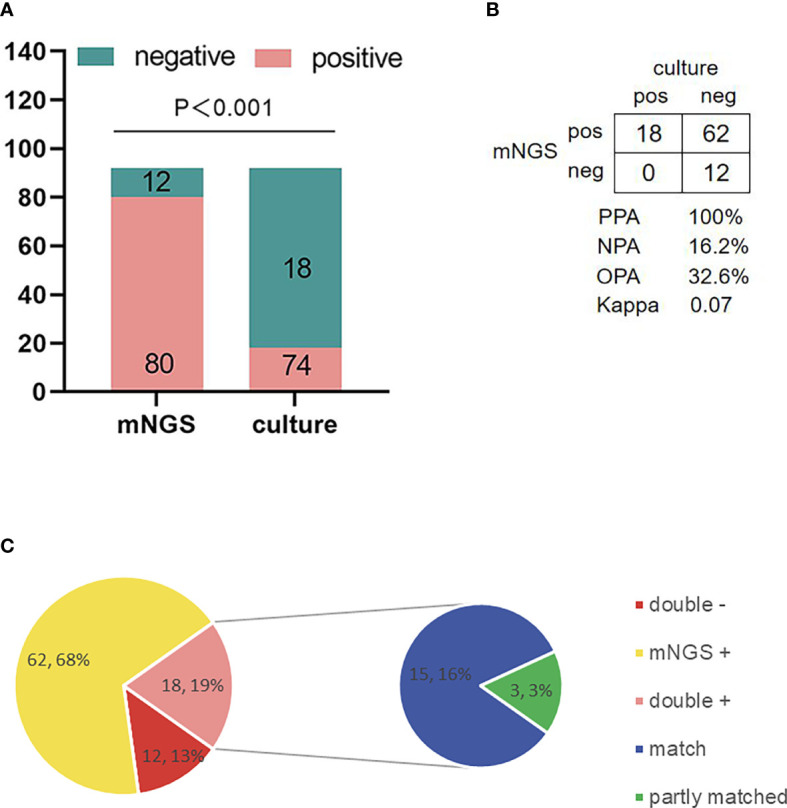
Comparison of the positive rate of metagenomic next-generation sequencing(mNGS) in spinal infection lesions and culture and analysis of the consistency of detection results. **(A)** The positive rate of mNGS was significantly higher than that of culture (*P* < 0.001). **(B)** Contingency tables formatted in a 2 × 2 manner showing mNGS and culture being formatted in the diagnosis of pathogens of spinal infection, because the positive rate of culture was low (18/92, 19.57%). The consistency between the two methods was poor (Kappa=0.07). **(C)** The pie chart shows the matching situation between mNGS and positive culture results. Among 18 positive culture samples, the culture results of 15 samples are completely consistent with mNGS. Three samples were partially consistent (at least 1 pathogen identified in the test was confirmed by the other), and there was no conflict between mNGS and culture results. mNGS, metagenomic next-generation sequencing; PPA, positive percent agreement; NPA, negative percent agreement; OPA, overall percent agreement.

The positive rate of microbial culture was 18/92 (19.57%), and the positive rate of mNGS detection was significantly higher than that of microbial culture (*X* ^2^=83.92, *P* < 0.001) (**Table 2**; **Figure 4A**). Eighteen samples were positive on both the microbial culture results and mNGS tests, and 12 samples were negative on both microbial culture results and mNGS tests. The positive percentage consistency was 100%, the negative percentage consistency was 16.2%, and the overall percentage consistency was 32.6%. Because of the low positive rate of culture, the consistency of the results of all the two assays was low (kappa=0.07) (**Figure 4B**). Among the 18 samples with positive cultures, the results of 15 samples were completely consistent with the results of mNGS detection, and the results of 3 samples were partially consistent with the results of mNGS detection (at least 1 pathogen identified in the test was confirmed by the other). In these 3 samples, the microbial culture results were used as the gold standard; the culture results of the first sample were *Streptococcus oralis*, and the mNGS results were *Streptococcus oralis* and *Streptococcus pneumoniae*. Culture results of the second sample were *Staphylococcus aureus*, and mNGS results were *Staphylococcus aureus* and *Human gammaherpesvirus 4*. The third sample was found to be *Escherichia coli* by culture, while mNGS was found to be *Mycobacterium abscessus* and *Escherichia coli*, mNGS detected multiple pathogens, but only one pathogen was cultured (**Figure 4C**).

### 3.5 Comparison of the positive rate of mNGS, microbial culture and X-pert against *Mycobacterium tuberculosis* of the spine

**Figure 5:**
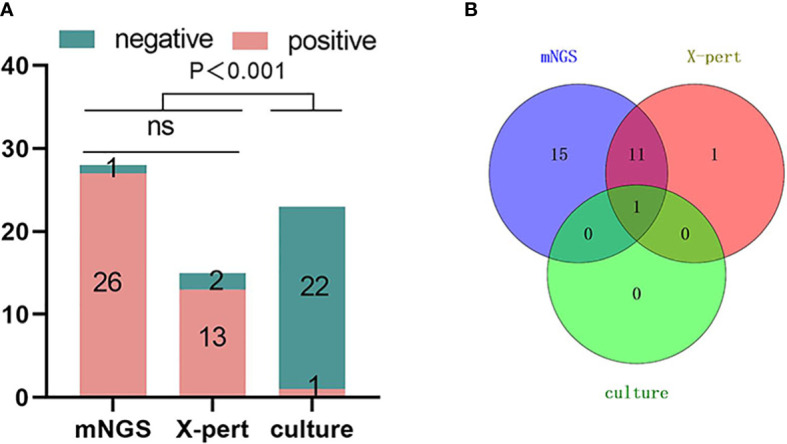
Comparison of mNGS, X-pert and culture positive rates for the detection of Mycobacterium tuberculosis in spinal tuberculosis focal tissue samples. The number of positive samples detected by the three detection methods showed in **(A)** that the positive rate of mNGS was higher than that of X-pert, but there was no statistical difference. The positive rate of mNGS and X-Pert was higher than that of culture, and the difference was statistically significant (P < 0.001). **(B)** Overlapping Venn diagram of samples positive for MTB by the three tests. mNGS, metagenomic next-generation sequencing; X-pert, the X-pert MTB/RIF assay; MTB, Mycobacterium Tuberculosis; ns, no significant difference.

The results of this study showed that among the 27 samples considered as spinal tuberculosis, 27 samples were tested by mNGS with 26 of the samples being positive by mNGS; 23 samples were cultured for mycobacterium with 1 positive; and 15 samples were tested with X-pert with 13 samples being positive. There was no significant difference in the detection rate of MTB between mNGS and X-pert (*P* > 0.05), but the detection rate of mNGS and X-pert was higher than that of culture (*P*<0.001) (**Figure 5A**). One sample was positive for MTB by the three detection methods, 11 samples were positive for MTB by mNGS and X-pert, 15 samples were positive for mNGS only, and 1 sample was positive for X-pert only (**Figure 5B**). 

### 3.6 Prognostic analysis of the two groups

**Figure 6:**
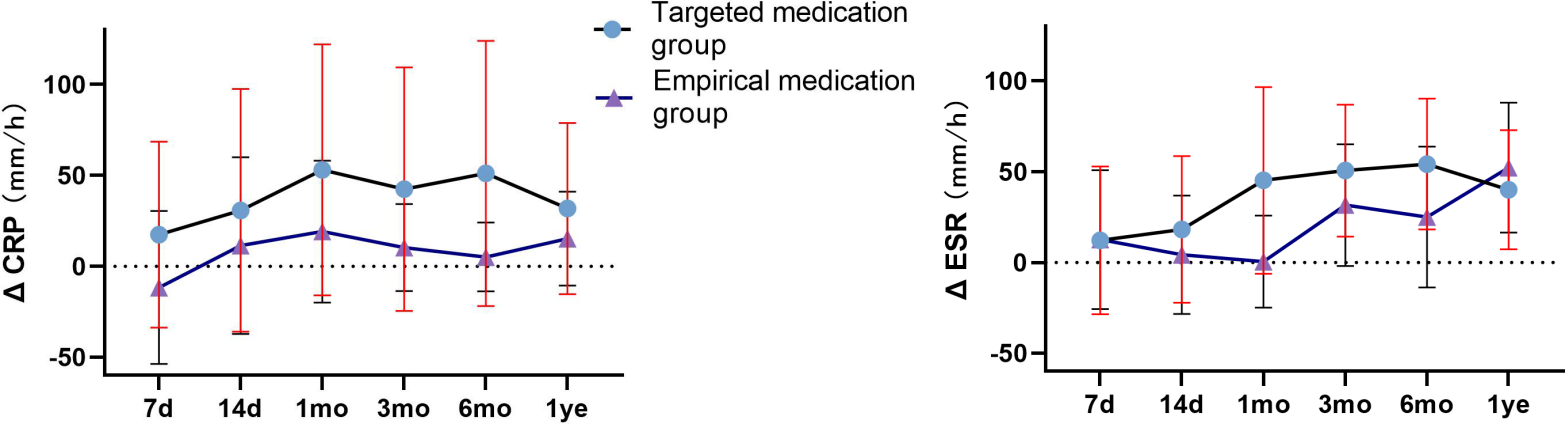
The comparison of the difference of ΔCRP (pre-op CRP-post-op CRP) and ΔESR (pre-op ESR-post-op ESR) between two groups at different follow-up times. CRP, C-reactive protein; ESR, erythrocyte sedimentation rate.

**Figure 7:**
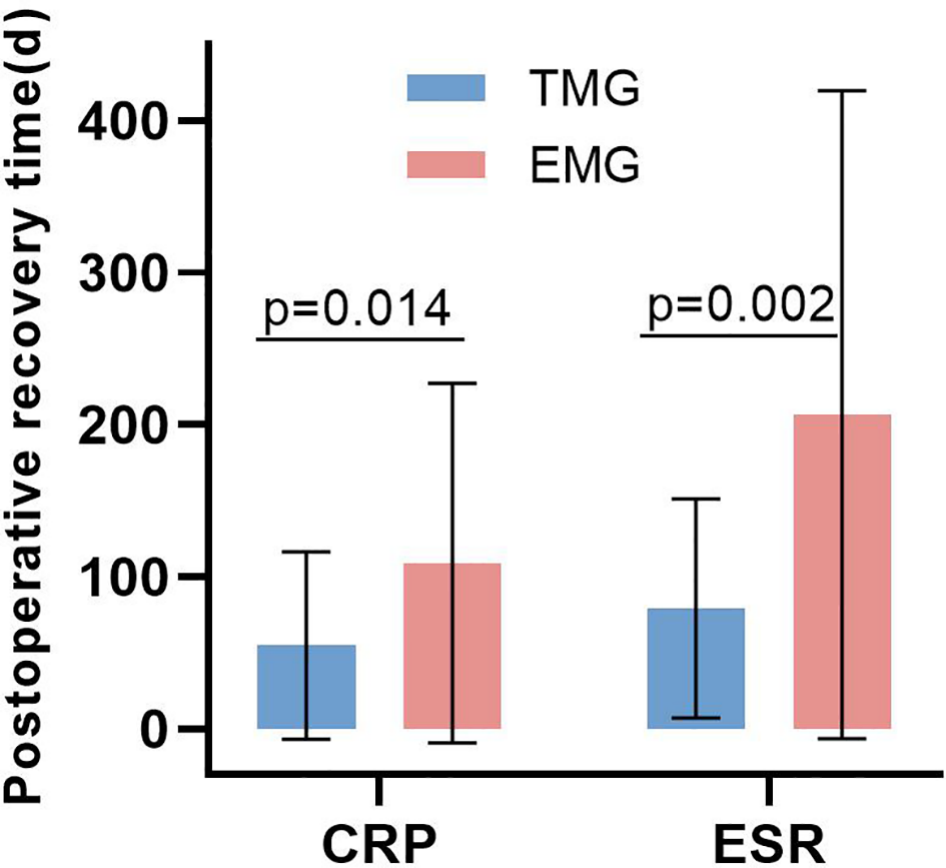
The comparison of the time for CRP and ESR to return to normal between the two groups. TMG, Targeted medication group; EMG, Empirical medication group.

**Figure 8:**
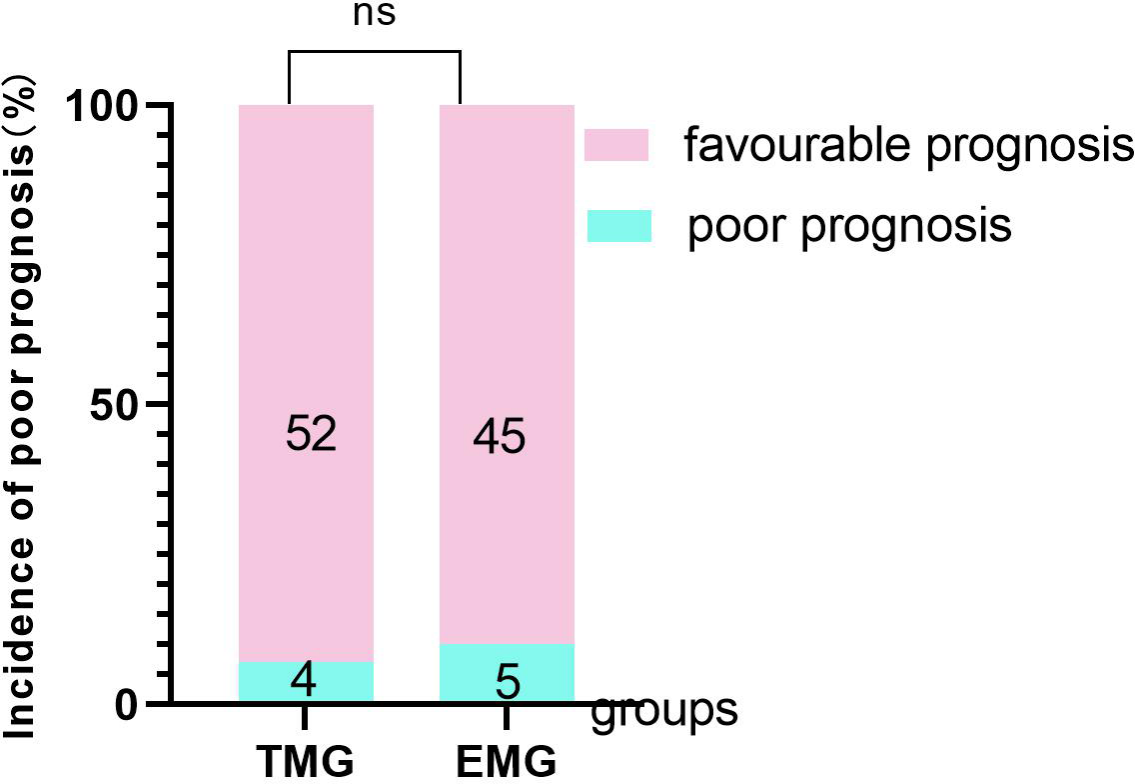
The comparison of the incidence of poor prognosis between the two groups. TMG, Targeted medication group; EMG, Empirical medication group.

CRP and ESR showed a downward trend in the two groups of patients with spinal infection. CRP and ESR in the targeted medication group showed a continuous downward trend, and the difference between ΔCRP (pre-op CRP—post-op CRP) at 7 days, 14 days, 3 months, and 6 months were significantly higher than that in the empirical medication group (*P* < 0.05). The difference between ΔESR (pre-op ESR—post-op ESR) in the targeted medication group at 1 month and 6 months was significantly higher than that in the empirical medication group (*P* < 0.05), and the difference was statistically significant (**Table 1**; **Figure 6**). The time required for CRP and ESR to return to normal after operation in the targeted drug group was significantly less than that in the empirical drug group (*P* < 0.05), and the difference was statistically significant (**Table 3**; **Figure 7**). A total of 106 patients completed the last follow-up. There were 9 patients with a poor prognosis observed postoperatively in the 4-patient medication group of patients who were in good condition after treatment, including 2 patients with poor wound healing and secondary debridement surgery, 1 patient with poor wound healing who underwent conservative treatment, and 1 patient with internal fixation of fracture, spinal internal fixation and removal. In the empirical medication group, 5 patients had a poor prognosis and were in good condition after treatment. Among them, 2 patients had poor wound healing and underwent secondary debridement surgery, 2 patients had poor wound healing and underwent conservative treatment, and 1 patient had recurrence of infection at the same surgical site after surgery. All patients had a significant effect of anti-infective treatment after the operation, and the clinical symptoms were improved. The incidence of poor postoperative prognosis in the treatment group was lower than that in the empirical treatment group, but there was no significant difference (*P* > 0.05) (**Table 3**; **Figure 8.**)

**Table 3:** The comparison of the time for CRP and ESR to return to normal and the incidence of poor prognosis between the two groups.

	Targeted medication group (n=80)	Empirical medication group (n=78)	*P*-value
Postoperative recovery time of CRP (d)(n,%) *	55.05±61.50 (40,50.0)	109.25±118.30 (38,48.7)	0.014
Postoperative recovery time of ESR (d)(n,%) †	79.37±72.00 (26,32.5)	206.77±213.06 (30,38.5)	0.002
Incidence of poor prognosis [n,(%)] ‡	4/56(4,7.1)	5/50(5,10.0)	0.737
poor wound healing	2	2	
Secondary debridement	1	2	
Fracture or loosening of internal fixation	1	0	
Recrudescence	0	1	

* one-way Wilcoxon rank-sum test: Z=2.455, P=0.14.

† one-way Wilcoxon rank-sum test: Z=3.090, P=0.002.

‡ one-way Fisher's exact test: P=0.737.

## 4 Discussion

To date, pathological examination is still the gold standard for diagnosing spinal infection, and microbial culture is the direct evidence of spinal infection (Bürger et al., 2020). However, the pathological examination cannot provide etiological information, and clinically, we found that culture has obvious limitations in the diagnosis of spinal infection pathogens, especially in the diagnosis of complex mixed infections in the spine, such as low detection rate and long time required for culture, which leads to diagnostic delay (Marschall et al., 2011; Kim et al., 2015; Pupaibool et al., 2015). For spinal surgeons, precision medicine has brought a problem. However, mNGS has high sensitivity and specificity in the diagnosis of pathogens of spinal infection, which is less affected by antibiotic exposure, does not rely on culture and unbiased extraction of all DNA for pathogen identification and typing and is less affected by the sample type (Miao et al., 2018). mNGS has significantly improved diagnosis of pathogens in spinal infections.

The results of this study showed that the positive rate of microbial culture in patients with spinal infection was 19.57%, which was significantly lower than the positive rate of mNGS detection, which was 86.96%. This may be related to the preoperative use of antibiotics (73 patients out of 92 patients who underwent mNGS and culture had preoperative use of antibiotics, the specific data are shown in **Supplement Data Table**). At the same time, we found a considerable number of rare pathogens, such as fungi and viruses, in the mNGS test results. Clinicians do not routinely screen and culture for these rare pathogens after surgery, which may also contribute to the low positive rate of cultures. We have 3 cases of mNGS results are not consistent with the result to cultivate, not included in this study, of which 1 patient mNGS results show that for the composite group of mycobacterium tuberculosis, culture result as *Escherichia coli*, but the patients with X - pert diagnosis of mycobacterium tuberculosis, so we consider culture specimen pollution, subsequent use anti-TB drug therapy, the prognosis is good. The mNGS results of another patient showed Candida parapsilosis, and the culture result was *Escherichia coli*. Considering the low positive rate of fungi in culture, we used amphotericin B combined with meropenem anti-infection treatment, and the patient had a good prognosis, but we could not determine which pathogen caused the infection in this patient. Another patient's preoperative blood culture results were *Staphylococcus aureus*, mNGS and culture results were negative, and the pathological results were considered infectious lesions. After our discussion, we decided to treat Staphylococcus aureus anti-infection, and the patient is in good condition. However, we cannot determine whether Staphylococcus aureus is the causative agent of the spinal infection in this patient.

In this study, it was found that mNGS could detect a variety of pathogens simultaneously, and the detection rate was high in the lesion tissue specimens of spinal mixed infection, while the detection rate of microbial culture for fungi, viruses and some rare pathogens was not ideal. Compared with traditional diagnostic methods, this study found that mNGS and X-pert had no significant difference in the detection rate of *Mycobacterium tuberculosis*, but they were significantly higher than that of *Mycobacterium tuberculosis* culture, which may be affected by the preoperative use of anti-tuberculosis drugs, resulting in a low positive rate of culture. The high incidence of spinal tuberculosis in this study may be related to the regional characteristics. China has always been a country with a high burden of tuberculosis, and the incidence of tuberculosis, especially spinal tuberculosis, is relatively high. People with spinal infections caused by Brucella usually live in livestock farming areas or have a history of exposure to an affected area. A proportion of our patients work in the livestock industry.

In addition, in this study, the positive rate of mNGS in diagnosing pathogens of spinal infection was significantly higher than that of microbial culture, but because the positive rate of microbial culture was low, it was not suitable to be used as a reference standard. Therefore, we used positive and negative percentage consistency to express the correlation between the two detection methods, but the consistency could not fully describe the performance of the detection methods. At present, most of the patients with spinal infection in our department are diagnosed with pathogens based on mNGS detection results in order to use antibiotics in a targeted way, and the majority of patients have achieved satisfactory curative effects. As shown in the results of this study, the time for CRP and ESR to return to normal in the patients in the targeted drug group was significantly less than that in the empirical drug group. In addition, we found in our study that, compared with the targeted use of antibiotics based on mNGS test results after surgery, the empirical use of antibiotics before surgery is mostly unreasonable. According to the 2015 Infectious Diseases Society of America (IDSA) Clinical Practice Guidelines for the Diagnosis and Treatment of Native Vertebral Osteomyelitis in Adults (Berbari et al, 2015), anti-infective treatment should be carried out for 4-6 weeks in most spinal infections of bacterial origin, and anti-infective treatment should be carried out for at least 3 months in patients with Brucella infection. When we adjusted the inflammatory indexes of the patients with postoperative follow-up based on the use of antibiotics, alternate use of antibiotics; the ESR and CRP of the patients return to normal; there were no local symptoms; oral antibiotics were used to maintain anti-infection treatment, according to our follow-up results; and most of the patients obtained satisfactory curative effect.

Peripheral blood CRP and ESR are commonly used to evaluate the prognosis of patients with spinal infection. In clinical practice, the prognosis of patients with spinal infection can be evaluated by regular follow-up review of inflammatory indicators such as CRP and ESR (Mavrogenis et al., 2017). In this study of spinal infection, the regular follow-up was used to collect data, and the statistical analysis found that the treatment group patients had CRP evaluated in the short-term follow-up within the postoperative follow-up of 6 months, but the CRP in the treatment group patients was significantly decreased. ESR in the treatment group was significantly decreased in comparison to the empirical medication group after being followed up for 1 month after surgery and after a postoperative follow-up of 6 months. Moreover, the recovery time of peripheral blood CRP in the two groups was significantly less than that of the peripheral blood ESR, indicating that the sensitivity of peripheral blood CRP is higher than that of peripheral blood ESR in the indicators reflecting the prognosis of patients with spinal infection. The recovery time of the peripheral blood CRP and ESR in the two groups was compared. The recovery time of CRP and ESR in the empirical medication group was significantly less than that in the empirical medication group, indicating that the patients with spinal infection in the treatment group had a faster decline in CRP and ESR in the short term compared with the patients with spinal infection in the empirical medication group. There was no significant difference in the decrease in CRP and ESR between the two groups at the 1-year postoperative follow-up, which may be because after a long period of postoperative antibiotic treatment and rehabilitation, most patients recovered to the normal range of CRP and ESR and basically achieved clinical cure. Our results show that the precise clinical use of antibiotics based on mNGS test results can help patients with spinal infection recover more quickly.

Some of the patients did not complete the last follow-up, and a small number of the patients were lost to follow-up for various reasons. However, the results of the two groups of patients with follow-up found that empirical use was associated with a higher postoperative prognosis for the treatment group, but the lack of statistical significance in the results may be the result of the fewer patients included.

Microbial culture plays a very important role in the diagnosis and treatment of spinal infection. The antibiotic susceptibility information provided by microbial culture is irreplaceable by other diagnostic methods, but mNGS can be used as an important supplement to culture to reduce the rate of missed diagnosis of spinal infection. At present, the cost of mNGS is still high, and there are still some limitations in routine use in clinical practice. For example, how to reduce the influence of human DNA and DNA from other foreign sources on the results, and how to determine whether the detected pathogens are pathogenic pathogens (Simner et al., 2018). But this is only temporary, with the progress of technology, these problems are gradually being overcome (Hasan et al., 2016). This is especially true for culture-negative spinal infection patients, as these detected mNGS data are important for exploring pathogen pathogenic genes and pathogenic mechanisms.

In conclusion, our results show that mNGS has a high positive rate in the detection of spinal infections, and targeted use of antibiotics according to the results of mNGS can make patients with spinal infections achieve a faster clinical cure. MNGS has good application prospects in the diagnosis and treatment of spinal infections.

## Data availability statement

The datasets involved in this study have been uploaded to the NCBI database, The Accession Numbers: PRJNA883025.

## Author contributions

GZ performed this study, analyzed the data, and wrote the manuscript; HZ, XH, DX, BT, MT, SL, and YL collected that data and performed statistical analysis; WX, metagenomic next-generation sequencing and quality control; QG designed the study and revised this paper. All authors contributed to the article and approved the submitted version.
